# COGNITIVE AND SUBJECTIVE EFFECTS OF OXYCODONE IN OLDER ADULTS WITH HEALTHY AND UNHEALTHY ALCOHOL PATTERNS

**DOI:** 10.1093/geroni/igz038.3256

**Published:** 2019-11-08

**Authors:** Monique Cherrier, Gregory Terman, Danny Shen, Laura Shireman, Andrew Saxon, Tracy Simpson, Preetma Kooner, Katie Wicklander

**Affiliations:** 1 University of Washington, Seattle, Washington, United States; 2 VA Puget Sound Health Care System, Seattle, Washington, United States

## Abstract

Chronic unhealthy levels of alcohol use, may predispose adults to use illicit substances and/or modify their response to prescribed medications, such as pain medications. We examined the cognitive and side effect response of older adults who met criteria for healthy and unhealthy alcohol drinking patterns after exposure to 10mg of oxycodone. Using a human laboratory model, eligible participants were characterized on cognitive, side effect measures and cold-pressor pain test (CPT) at baseline and repeated 90 minutes, 3 and 5 hours post dosing (10mg oxycodone). Blood samples were taken at regular intervals to measure drug levels. One-hundred twenty-five adults completed the study day, eighty participants with heavy alcohol consumption and 45 with healthy. Middle age (MA) group had a mean age of 51 (11.2) years, older adults (OA) 72 (4.2) years. Between group (unhealthy vs healthy drinkers, middle age vs older adult) comparisons for cognitive performance indicate a significant decline at 90 min. However, MA and OA heavy alcohol consumers evidenced less decline on sustained attention (D2) and working memory, but more decline on a measure of balance (berg). Anti-nocioceptive effects were greatest in healthy (MA,OA) in comparison to heavy, however there were no differences on pupil miosis. Subjective rating of side effects were rated more severe in the OA unhealthy group compared to MA and healthy. These findings indicate unhealthy alcohol consumption attenuates the impact of opioid medication. Results indicate that alcohol consumption patterns should be considered when using opioids in older and middle age adults.

